# Yeast Survive by Hedging Their Bets

**DOI:** 10.1371/journal.pbio.1001327

**Published:** 2012-05-08

**Authors:** Robin Meadows

**Affiliations:** Freelance Science Writer, Fairfield, California, United States of America

## Abstract

A new experimental approach reveals a bet hedging strategy in unstressed, clonal yeast cells, whereby they adopt a range of growth states that correlate with expression of a trehalose-synthesis regulator and predict resistance to future stress.

**Figure:**
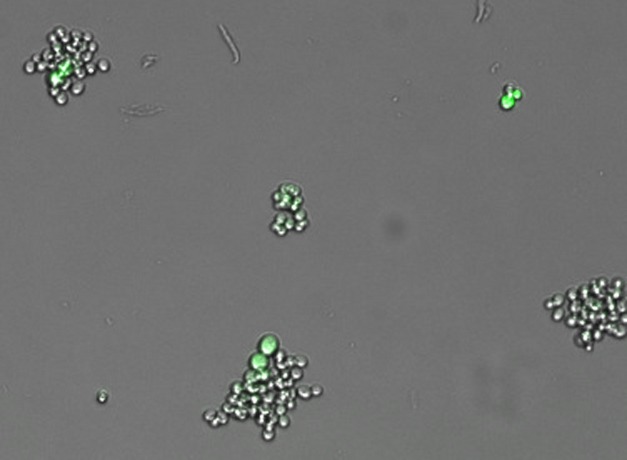
Bet hedging in yeast is revealed by a new assay that measures variation in growth rate, fluorescent-protein expression, and stress survival in tens of thousands of tiny colonies simultaneously.

Investing in opposite outcomes—or bet hedging—is a common tool to cushion against huge monetary losses. While this strategy has earned a bad name for its role in the recent global financial crisis, bet hedging is key to survival in bacteria. Populations of genetically identical bacteria include both fast- and slow-growing cells. The former flourish during good times, while the latter hedge against future bad times, capable of surviving stresses that kill off the rest of the population. How microorganisms manage bet hedging at the molecular level is poorly understood. Now, in this issue of *PLoS Biology*, Sasha Levy, Naomi Ziv, and Mark Siegal shed light on the mechanism. Working with the yeast *Saccharomyces cerevisiae*, they show that slow-growing cells resist stress better than fast-growing cells, thanks in part to higher levels of a stress-related protein.

Bet hedging in microorganisms is important not just for the microbes but has implications for humans as well. When the bacterium *Escherichia coli* is treated with antibiotics, most of the population dies rapidly. But the cells that grow more slowly survive the treatment longer, and can switch to fast growth once the antibiotic is removed. Such slow-growing “persister” cells benefit microorganisms but potentially complicate treatments for infection. A similar situation may obtain in cancer; melanomas contain rare cells that divide slowly, thus evading chemotherapies that target fast-dividing cells, and these slow-dividing cells can later give rise to daughter cells that proliferate at great rates.

Levy and colleagues discovered bet hedging in yeast with a new assay they developed, which measures factors including growth rate, protein expression, and survival in tens of thousands of tiny yeast colonies simultaneously. When the researchers grew colonies from single cells in a genetically identical population, they found that up to a tenth of the colonies were slow growers. This suggested that, as in bacteria, rapidly growing yeast populations also have slow-growing cells in the mix that might serve as a hedge against future challenges. However, while bacterial cells have been characterized as simply switching between two fixed growth states—slow versus fast—the yeast colonies displayed a continuous range of growth states from very slow to very fast.

Screening for gene expression associated with bet hedging revealed that slow-growing yeast also had higher levels of a protein (Tsl1) needed to synthesize trehalose, a sugar thought to confer resistance to stresses including heat, freezing, dessication, and alcohol. Moreover, abundance of this protein varied continuously across the range of growth rates: the slower the growth, the higher the Tsl1 level. Importantly, progeny of either fast- and slow-growing cells could ultimately reproduce the population's ancestral growth-rate continuum, showing that, like bacteria, yeast can switch between growth states.

Besides growing slowly, cells with high Tsl1 levels were more resilient when stressed and, intriguingly, tended to be on the old side. Tsl1-rich cells, like old cells, were often bigger and bore more of the bud scars that come from repeated cell divisions. To test stress resistance, the researchers subjected yeast to heat shock, and found both that survival was higher in slow growers and that Tsl1 contributes directly to their resilience. When subjected to temperatures high enough to make an induced response irrelevant, survival was still higher in wild-type yeast than in a Tsl1-deficient mutant.

This work suggests that bet hedging in yeast is more complex than the scenario commonly accepted for bacteria. Not only does yeast have a continuous range of growth states rather than the simple fast-versus-slow found in bacteria, but switching between fast and slow growth states is thought to be stochastic in bacteria. In contrast, the link between age and stress resistance in yeast suggests that more deterministic factors may also be at play in this eukaryote. Similarly, bet hedging is likely to be complex in human cancers. If, as in yeast, malignant cells switch between multiple growth states, the researchers' new findings could help unravel the bet hedging mechanisms that contribute to the failure of chemotherapies over the long term.

**Levy SF, Ziv N, Siegal ML (2012) Bet Hedging in Yeast by Heterogeneous, Age-Correlated Expression of a Stress Protectant. doi:10.1371/journal.pbio.1001325**

